# Delayed presentation of bowel obstruction after abdominal blunt trauma

**DOI:** 10.1016/j.tcr.2021.100414

**Published:** 2021-02-10

**Authors:** M. Shalhoub, F. Alghamdi, F. Alsannaa

**Affiliations:** a General Surgery Department, Prince Sultan Military Medical City, Riyadh, Saudi Arabia; b King Saud University, Trauma & Acute Care Fellow, Riyadh, Saudi Arabia

**Keywords:** Abdominal trauma, Obstruction, Blunt trauma, Delayed presentation

## Abstract

Blunt abdominal trauma is a rare cause of small bowel obstruction thought to arise from either a sealed perforation of the small bowel or mesenteric injury resulting in adhesions. A 55-year-old gentleman presented with symptoms and signs of small bowel obstruction and a history of blunt abdominal trauma 14 months previously. Abdominal computed tomography showed a transition zone at the terminal ilium with proximal dilatation indicative of obstruction. At surgery, he had adhesions involving the terminal ilium with shortening and fibrosis of the supplying mesentery. Patients with a history of blunt abdominal trauma presenting with abdominal symptoms must be investigated to rule out bowel obstruction, with a low threshold for surgical intervention.

## Introduction

Adhesions, masses, and hernias account for >80% of presentations of small bowel obstruction, with other less common causes including volvulus, intussusception, and inflammatory bowel disease [1]. Blunt abdominal trauma is a rare cause of small bowel obstruction that might be caused by mesenteric injury leading to ischemia, fibrosis, and subsequent obstruction or micro-perforation of the intestinal wall after trauma that heals by fibrosis and luminal stenosis [2]. Here we present a case small bowel obstruction occurring after blunt abdominal trauma to revisit the pathophysiology of traumatic small bowel obstruction and to remind clinicians of the diagnostic criteria and the role of surgical intervention in these cases.

## Case report

A 55-year-old gentleman with no history of abdominal surgery presented to the emergency department with a two-day history of sudden onset, colicky abdominal pain of increasing intensity and abdominal distention. He had nausea and vomiting but there was no history of fever, rectal bleeding, or melena. On examination there was tenderness in the lower left quadrant but no peritoneal signs, and he was clinically stable.

His history from another hospital reported trauma 14 months previously after being hit as a pedestrian by a car. He had sustained multiple facial and orthopedic injuries, which had been managed by the orthopedic team. Computed tomography (CT) of the abdomen at that time showed a small gastrocolic ligament hematoma, right adrenal hematoma, sub-capsular liver hematoma, and small sigmoid mesocolon contusions, which were managed conservatively. The patient was discharged after seven weeks in hospital. He had sought medical advice one month after discharge due symptoms and signs of bowel obstruction, with an abdominal CT performed at that time showing distal ileal obstruction with luminal narrowing and a transition zone suggestive of an adhesive band with surrounding fat stranding and mild intestinal wall thickening. The patient improved with conservative management and he was discharged after resolution of the obstructive symptoms.

**Fig. 1:**
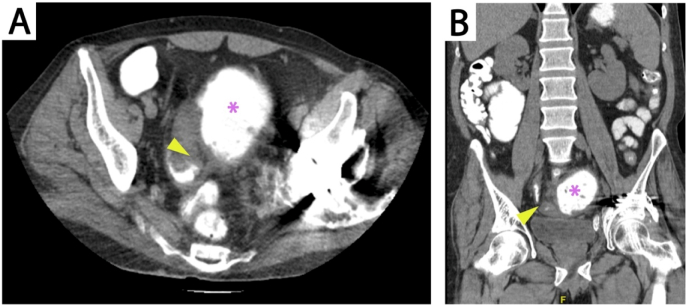
Abdominal oral contrast CT showing a transition zone at the terminal ilium (arrow) and proximal bowel dilatation (asterisks) in axial (A) and coronal (B) views.

**Fig. 2:**
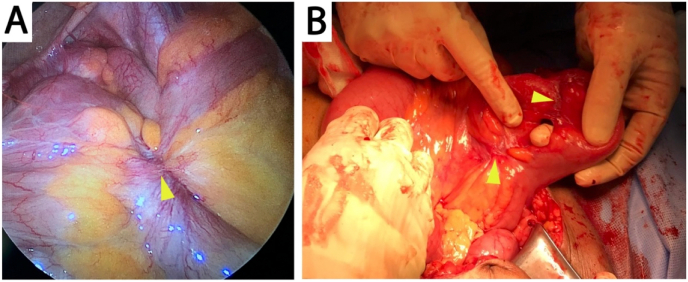
Laparoscopic view (A) showing mesenteric scarring (arrow) with adhesions involving the terminal ilium, with the laparotomy view (B) showing adhesions involving two terminal ileal loops causing partial closed-loop obstruction.

**Fig. 3:**
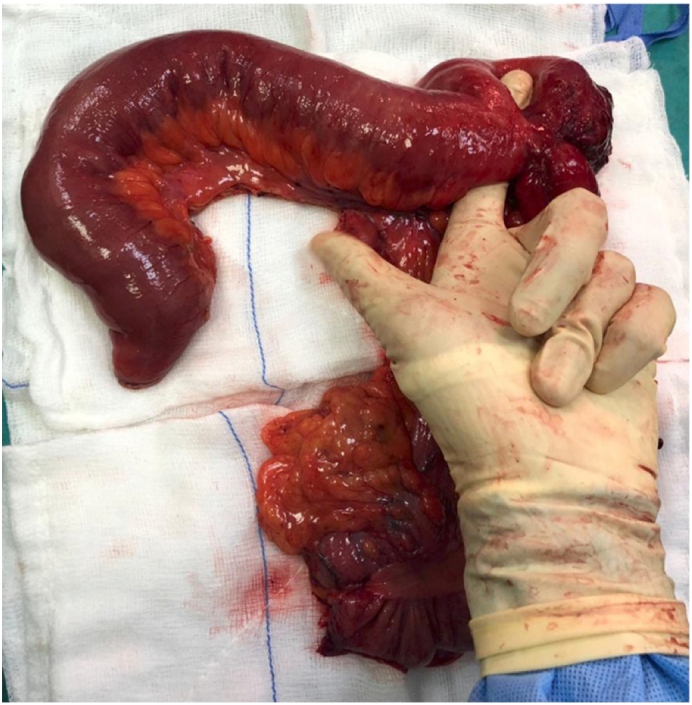
Resection specimen showing dense, inseparable adhesions attached to two lumens of the distal ilium.

During his current admission, tumor markers were negative. Abdominal X-ray revealed dilated intestinal loops and multiple air-fluid levels, and an abdominal CT with oral contrast again showed luminal narrowing with a transition zone at the terminal ilium and proximal dilated bowel loops with contrast passing distally (Fig. 1). The patient underwent laparoscopic exploration, at which there was ileocecal mesenteric scarring and shortening with extensive adhesions involving the terminal ilium to cause a closed loop partial obstruction (Fig. 2, Fig. 3). The procedure was converted to open laparotomy, and an ileocecal resection with primary anastomosis was performed. Histopathological examination of the resection specimen showed small bowel mucosa with active chronic inflammation with surface ulceration and granulation tissue formation, cryptitis and fissure formation, and serosal fibrosis. The patient was discharged after two weeks after an uneventful hospital course and was followed-up in clinic for two months without exhibiting any signs or symptoms of recurrent obstruction.

## Discussion

Although small bowel injury after blunt abdominal trauma is rare, small bowel obstruction can occur after blunt abdominal trauma and account for 1% of small bowel obstruction cases [3]. The presentation can be delayed, occurring weeks to months after trauma [4].

One suggested mechanism of bowel obstruction after blunt abdominal trauma is mesenteric injury leading to ischemia of the supplied bowel segment and subsequently fibrosis and stenosis of the involved segment [5]; this may have been a contributing factor in our case, since mesenteric scarring and fibrosis of the ileocecal mesentery were present. Another suggested mechanism is micro-perforation of the bowel from blunt abdominal trauma, with a higher risk of such injuries in bowel segments with shorter and relatively fixed mesenteries, particularly the proximal jejunum and terminal ilium [6]. These micro-perforations usually seal immediately after trauma and heal through fibrosis, which might later cause adhesive obstruction; this may have also contributed to the obstruction in our case. In addition, midline bowel segments such as the duodenojejunal flexure and transverse and sigmoid colon are also prone to blunt injury as they can be compressed against the spine [6].

The first CT at the time of the accident showed no significant bowel or mesenteric injury; however, he presented one and 14 months later with bowel obstruction and CT findings of distal ileal narrowing with a transition zone. Given the intra-operative finding of distal ileal adhesions associated with mesenteric fibrosis and scarring and intraabdominal contusions and hematomas noted at the time of the accident, the most likely cause of the obstruction was mesenteric injury at the time of trauma that led to ischemia of the supplied bowel segment and subsequent luminal stenosis. These specific mesenteric injuries can be missed during the first presentation after trauma and can be difficult to diagnose or even misdiagnosed as suspected malignancy or inflammatory bowel disease when presenting later with bowel obstruction [7]. Hence, and as the bowel wall in such cases is irreversibly stenotic, patients are less likely to improve with non-operative management and surgical exploration is indicated with either an open or laparoscopic procedure in selected cases where there is a clear cause of obstruction [7]. In our case, we started with diagnostic laparoscopy but, due to the extensive adhesions and need to explore the abdomen further, we elected to convert to an open operation with resection of the involved terminal ilium and cecum. Our patient recovered without complications.

Patients with a history of abdominal trauma and symptoms of bowel obstruction should preferably be investigated with contrast-enhanced abdominal and pelvic CT scans and later colonoscopy [8]. Criteria proposed for the diagnosis of bowel obstruction after blunt abdominal trauma include the appropriate history, no similar attacks before the trauma, onset of symptoms after the trauma, confirmation of bowel stenosis, and exclusion of other specific neoplastic or inflammatory diseases in the related bowel segment [3], all of which were applicable in our case.

In conclusion, patients with abdominal symptoms suggestive of bowel obstruction and a history of abdominal trauma should be thoroughly investigated, with a lower threshold for surgical intervention.
